# [^18^F]FDG-PET/CT radiomics for the identification of genetic clusters in pheochromocytomas and paragangliomas

**DOI:** 10.1007/s00330-022-09034-5

**Published:** 2022-08-24

**Authors:** Wyanne A. Noortman, Dennis Vriens, Lioe-Fee de Geus-Oei, Cornelis H. Slump, Erik H. Aarntzen, Anouk van Berkel, Henri J. L. M. Timmers, Floris H. P. van Velden

**Affiliations:** 1 Department of Radiology, Section of Nuclear Medicine, Leiden University Medical Center, Leiden, the Netherlands; 2 TechMed Centre, University of Twente, Enschede, the Netherlands; 3 Department of Medical Imaging, Radboud University Medical Center, Nijmegen, the Netherlands; 4 Division of Endocrinology, Department of Internal Medicine, Radboud University Medical Center, Nijmegen, the Netherlands

**Keywords:** Pheochromocytomas, Mutation, [^18^F]FDG-PET/CT, Logistic regression, AUC

## Abstract

**Objectives:**

Based on germline and somatic mutation profiles, pheochromocytomas and paragangliomas (PPGLs) can be classified into different clusters. We investigated the use of [^18^F]FDG-PET/CT radiomics, SUV_max_ and biochemical profile for the identification of the genetic clusters of PPGLs.

**Methods:**

In this single-centre cohort, 40 PPGLs (13 cluster 1, 18 cluster 2, 9 sporadic) were delineated using a 41% adaptive threshold of SUV_peak_ ([^18^F]FDG-PET) and manually (low-dose CT; ldCT). Using PyRadiomics, 211 radiomic features were extracted. Stratified 5-fold cross-validation for the identification of the genetic cluster was performed using multinomial logistic regression with dimensionality reduction incorporated per fold. Classification performances of biochemistry, SUV_max_ and PET(/CT) radiomic models were compared and presented as mean (multiclass) test AUCs over the five folds. Results were validated using a sham experiment, randomly shuffling the outcome labels.

**Results:**

The model with biochemistry only could identify the genetic cluster (multiclass AUC 0.60). The three-factor PET model had the best classification performance (multiclass AUC 0.88). A simplified model with only SUV_max_ performed almost similarly. Addition of ldCT features and biochemistry decreased the classification performances. All sham AUCs were approximately 0.50.

**Conclusion:**

PET radiomics achieves a better identification of PPGLs compared to biochemistry, SUV_max_, ldCT radiomics and combined approaches, especially for the differentiation of sporadic PPGLs. Nevertheless, a model with SUV_max_ alone might be preferred clinically, weighing model performances against laborious radiomic analysis. The limited added value of radiomics to the overall classification performance for PPGL should be validated in a larger external cohort.

**Key Points:**

• *Radiomics derived from [*^*18*^*F]FDG-PET/CT has the potential to improve the identification of the genetic clusters of pheochromocytomas and paragangliomas.*

• *A simplified model with SUV*_*max*_ *only might be preferred clinically, weighing model performances against the laborious radiomic analysis.*

• *Cluster 1 and 2 PPGLs generally present distinctive characteristics that can be captured using [*^*18*^*F]FDG-PET imaging. Sporadic PPGLs appear more heterogeneous, frequently resembling cluster 2 PPGLs and occasionally resembling cluster 1 PPGLs.*

**Supplementary Information:**

The online version contains supplementary material available at 10.1007/s00330-022-09034-5.

## Introduction

Pheochromocytomas and paragangliomas (PPGLs) are rare catecholamine-producing neuroendocrine tumours arising from the chromaffin cells in the adrenal medulla and extra-adrenal sympathetic ganglia [1]. In around 40% of patients, PPGLs are part of hereditary syndromes caused by germline mutations [2]. Germline mutations in at least a dozen PPGL susceptibility genes with relevant prevalence have been identified, including VHL (von Hippel-Lindau), SDHA/B/C/D/AF2 (succinate dehydrogenase subunits A, B, C, D and assembly factor 2), RET (rearranged during transfection), NF1 (neurofibromatosis type 1), MAX (myc-associated factor X) and TMEM127 (transmembrane protein 127) [3–5]. Moreover, in 30–40% of sporadic PPGLs, somatic mutations are identified [5]. Based on both germline and somatic mutations, PPGLs can be classified into two clusters: cluster 1 PPGLs, characterised by increased expression of genes involved in hypoxia (i.e. SDHx/VHL), and cluster 2, associated with kinase signalling (i.e. RET/NF1) [6, 7]. The characterisation of the genetic cluster is of interest, because the underlying mutations directly impact the clinical presentation of PPGLs. The prevalence of malignancy generally varies between 10 and 17%, but reaches even 40% in patients with *SDHB* mutations [8].

Genetic differences result in phenotypic differences with regard to cellular metabolism, which can be observed in the uptake of 2-[^18^F]fluoro-2-deoxy-D-glucose ([^18^F]FDG) by positron emission tomography (PET), with relatively high standardised uptake values (SUV) detected in cluster 1 PPGLs [9, 10]. Previous research in a part of this cohort showed that the maximum SUV (SUV_max_) could distinguish hereditary cluster 1 and 2 PPGLs with an area under the receiver operating characteristic curve (AUC) of 0.91 (95% CI: 0.80–1.00) [11]. In addition to the traditional quantitative PET features such as the SUV_max_, radiomics allows quantification of tracer uptake heterogeneity and other imaging features [12, 13]. Radiomics, the extraction of large amounts of quantitative features from medical imaging, aims to find stable and clinically relevant image-derived biomarkers that provide a non-invasive way of quantifying and monitoring disease characteristics in clinical practice [14]. Literature on radiomics in PPGLs is scarce, but includes a computed tomography (CT) approach for the differentiation between pheochromocytomas and lipid-poor adenoma [15], an approach on T2 weighted fat-saturated magnetic resonance imaging for the differentiation of paragangliomas from other neck masses [16] and a PET approach studying characterisation of the genetic cluster in pheochromocytomas [17].

This study investigated the potential utility of radiomic features derived from PET and low-dose CT for the characterisation of the genetic cluster of PPGLs.

## Materials and methods

### Patient population

Patients with PPGL with known mutation status and who underwent a [^18^F]FDG-PET/CT scan in the Radboud University Medical Center between 2011 and 2018 were retrospectively included. A selection of these patients has previously been studied [10, 11, 18]. This retrospective database study has been reviewed and approved by the Commission on Medical Research Involving Human Subjects Region Arnhem-Nijmegen, the Netherlands. Informed consent was waived because of the retrospective nature of the study. Patients that objected to the use of their anonymised data were excluded.

All patients underwent genetic testing for germline mutations in known susceptibility genes (SDHA/B/C/D/AF2, RET, VHL, TMEM127 and MAX) using standard clinical diagnostic procedures. In case no germline mutation was found, somatic mutations were obtained from post-operative histology. The classes cluster 1 and 2 contain both germline and somatic mutations. The class sporadic contains sporadic PPGLs without known mutations found in germline and tumour tissue associated with cluster 1 or 2. The biochemical diagnosis was based upon the collection of plasma-free metanephrines (metanephrine, normetanephrine and 3-methoxytyramine; metabolites of the catecholamines adrenaline, noradrenaline and dopamine, respectively) and assayed using high-performance liquid chromatography or liquid chromatography–mass spectrometry [19]. Patients were excluded when no [^18^F]FDG-PET/CT scan was acquired (*N* = 40) and when patients without a germline mutation were not tested for a somatic mutation (*N* = 33).

### Data acquisition and image reconstruction

PET/CT images were acquired in accordance with the European Association of Nuclear Medicine (EANM) guidelines version 1.0 for tumour PET imaging [20] using a Biograph 40 mCT (Siemens Healthineers). Patients fasted for at least 6 h and serum glucose levels were below 8.0 mmol/L. Image acquisition was started 60 (55–75) minutes after intravenous administration of [^18^F]FDG. The reconstructed voxel sizes were 3.18 × 3.18 × 3.00 mm^3^ for PET and ranged from 0.64 × 0.64 × 3 mm^3^ to 1.27 × 1.27 × 3 mm^3^ for non-contrast-enhanced low-dose (ld) CT images.

Additional details on data acquisition, image reconstruction, radiomic analysis and the statistical analysis can be found in Online Resource 1: the Image Biomarker Standardisation Initiative (IBSI) Supplementary File 1 [21], which also includes the TRIPOD statement (transparent reporting of a multivariable prediction model for individual prognosis or diagnosis, version October 1, 2020) [22].

### Quantitative image analysis

#### Volume of interest (VOI) delineation

**Fig. 1:**
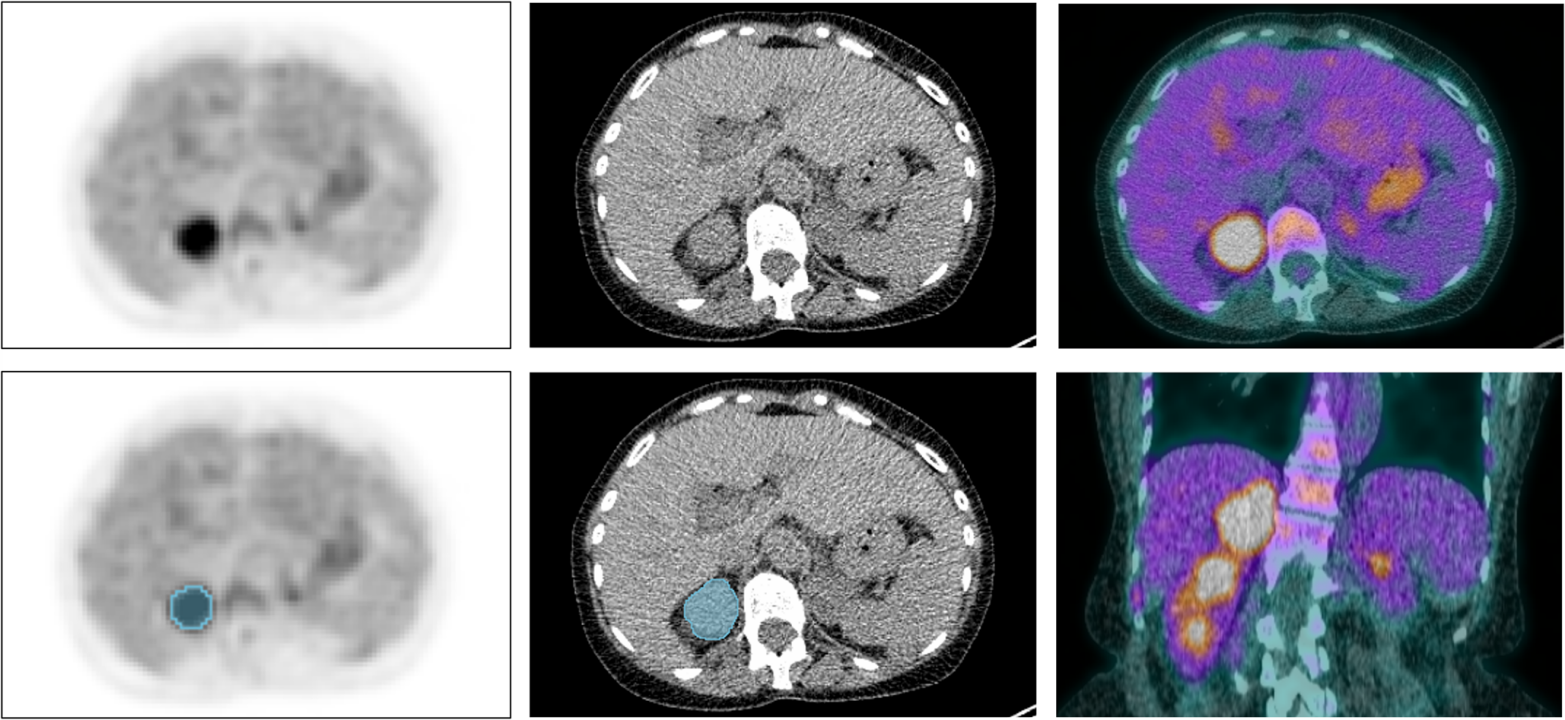
[^18^F]FDG-PET/CT scan of patient with a sporadic pheochromocytoma in the right adrenal. The blue area denotes the volume of interest, SUV_max_ = 7.16 g/mL, SUV_mean_ = 4.76 g/mL, metabolic tumour volume = 25.97 cm^3^

VOI delineation was performed in 3DSlicer version 4.11 (www.slicer.org) [23] and in-house built software implemented in Python 3.7 (Python Software Foundation). Boxing was applied to exclude surrounding [^18^F]FDG-avid tissues like the kidneys or catecholamine-stimulated brown adipose tissue. Since [^18^F]FDG uptake of PPGLs can be rather low and heterogeneous, PPGLs were delineated using an isocontour that applies an adaptive threshold of 41% of the SUV_peak_, obtained using a sphere of 12 mm diameter [24], corrected for local background (Fig. 1, more details in Supplementary File 1) [25]. This method demonstrated the best agreement between delineated tumour sizes and pathological tumour sizes [26] and allowed the inclusion of most of the vital tumour volume, while minimising the need for boxing. Regions of central necrosis, which were not included by the adaptive threshold algorithm, were not manually added to the VOI, since in PPGL, the classification performance of the radiomic model is not affected by the addition of areas of central necrosis [18]. LdCT images were delineated manually. Lesions were excluded when edges could not be distinguished from intense brown adipose tissue activation (*N* = 2) [27] or when the minimal size recommendation of 64 voxels per VOI was not met (*N* = 1) [28].

#### Image processing

LdCT voxels were interpolated to isotropic voxels (1.5 × 1.5 × 1.5 mm^3^) using B-spline interpolation, with grids aligned by the input origin. PET images were not interpolated, since the voxels were almost isotropic (3.18 × 3.18 × 3.00 mm^3^).

#### Radiomic feature extraction

Radiomic feature extraction was performed in PyRadiomics 3.0 in Python 3.7 (Python Software Foundation) [29]. For both PET and ldCT images, 105 radiomic features were extracted: 18 first-order features, 14 shape features and 73 texture features (Supplementary File 1). In addition, the total lesion glycolysis, the product of the mean SUV and the metabolic tumour volume, was calculated. A fixed bin size of 0.5 g/mL and 25 Hounsfield units was applied for PET and CT images, respectively.

### Statistical analysis

**Fig. 2:**
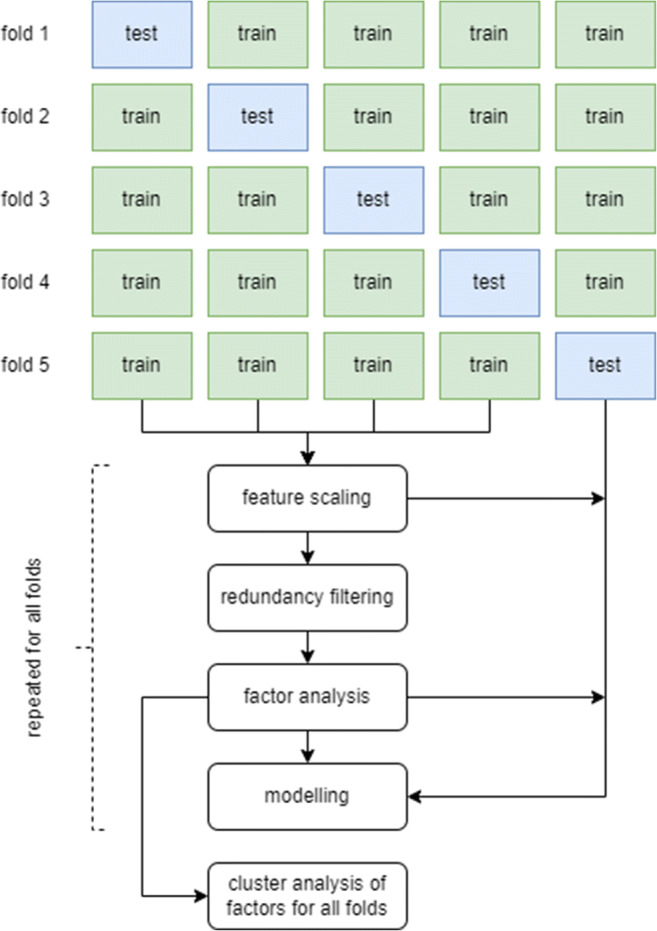
Schematic overview of statistical analysis, consisting of stratified 5-fold cross-validation with dimensionality reduction incorporated in the folds. Per fold, scaling was performed (centred around 0, variance of 1), followed by redundancy filtering of the Spearman correlation matrix (*ρ* = 0.95) and factor analysis using an orthogonal rotation. The factor definitions were determined based on the underlying clusters of features in the different folds

Stratified five-fold multinomial logistic regression for the identification of the genetic clusters of PPGLs was performed in R version 3.6.0 (R Foundation for Statistical Computing). Heatmaps were generated using Orange Data Mining version 3.30.2 (University of Ljubljana) [30]. The dataset was split into five equal-sized folds, stratified for the genetic clusters. Each subgroup consecutively served as a test set and the remaining four-fifths of patients served as the training set. Per fold, dimensionality reduction of the radiomic feature set in the training set was performed using redundancy filtering and factor analysis in FMradio (Factor Modelling for Radiomics Data) R-package version 1.1.1 (Fig. 2) [31]. One feature was selected for every ten subjects in the training set [14]. Features were scaled (centred around 0, variance of 1) to avoid that features with the largest scale would dominate the analysis. Redundancy filtering on the Spearman correlation matrix (*ρ* = 0.95) of the scaled features was performed. Factor analysis was performed on the redundancy filtered correlation matrix using an orthogonal rotation, so that the first factor explained the largest possible variance in the dataset and succeeding factors explained the largest variance in orthogonal directions. The sampling adequacy of the model was determined by the Kaiser-Meier-Olkin measure (KMO, ≥ 0.9) [31]. The factor definitions were determined based on the underlying clusters of features in the different folds. Models were trained for the SUV_max_, PET(/CT) factors and imaging variables combined with the biochemical profile. Catecholamines are included as separate dichotomous variables (e.g. adrenergic: yes/no). In addition, the best-performing factor-based model was approximated by a feature-based model using the features underlying the factors, to advance the reproducibility of the radiomic model. The imaging factors and features and the biochemical profile were used as independent variables in multinomial logistic regression. Classification performances were presented as mean multiclass AUCs and mean AUCs between clusters as determined over the five folds for the test sets [32]. A sham experiment was conducted to validate the findings [33]. The outcome labels were randomly shuffled for 100 iterations and mean AUCs were calculated. Randomisation of the outcome labels preserves the distributions and multicollinearity of the radiomic features and the prevalence of the outcome, but it uncouples their potential relation.

## Results

Forty PPGLs in 38 patients were analysed, including thirteen cluster 1, eighteen cluster 2 and nine sporadic PPGLs (Table 1).

**Table 1:** Clinical characteristics of 40 PPGLs (38 patients). *SUV* standardised uptake value

Characteristic	Value
Age (years), median (range)	50 (23–89)
Sex (M/F)	17/21
Tumour location	
• Pheochromocytoma • Paraganglioma	34 6
Genetic cluster	
• Cluster 1 (germline/somatic) • Cluster 2 (germline/somatic) • Sporadic	10/3 10/8 9
Biochemical profile^a^	
• Adrenergic • Noradrenergic • Dopaminergic	17 24 6
SUV_max_ (g/mL), median (range)	4.75 (1.67–36.01)
Metabolic tumour volume (cm^3^), median (range)	29.07 (2.28–253.86)

^a^Combinations of biochemical profiles occur

**Fig. 3:**
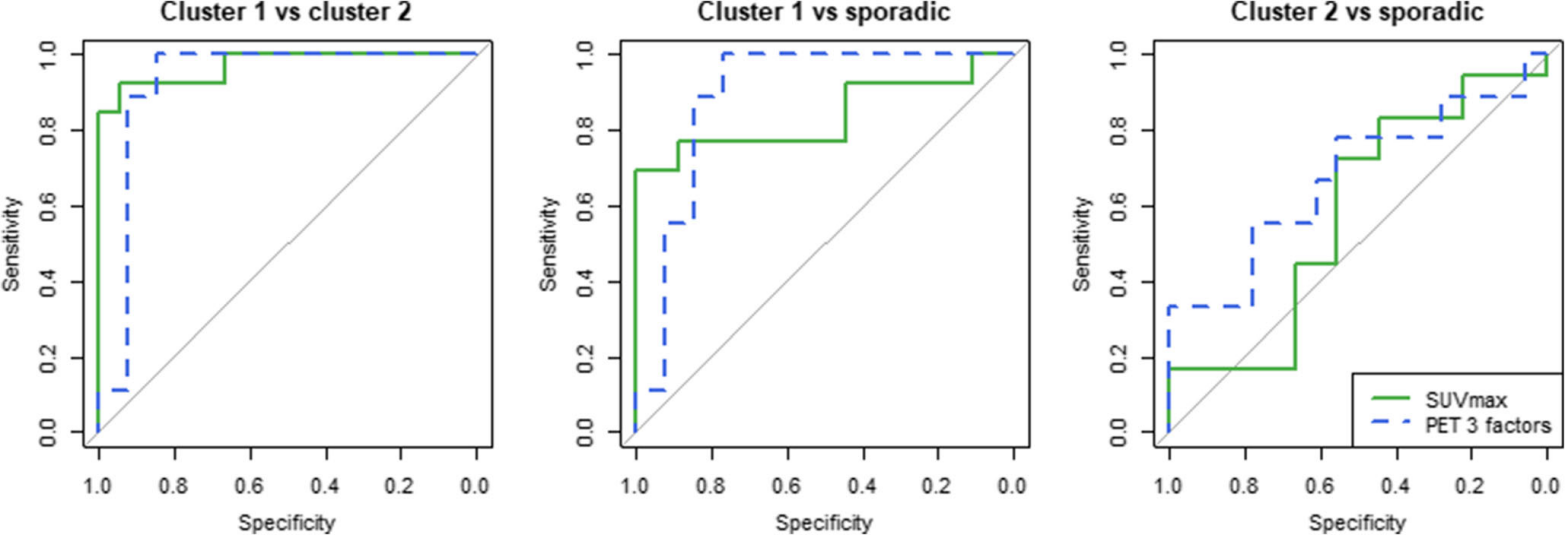
ROC curves for the SUV_max_ (green, solid) and PET three-factor model (blue, dashed) between clusters (cluster 1 vs 2, cluster 1 vs sporadic, cluster 2 vs sporadic) as determined by stratified five-fold multinomial logistic regression and the multiclass AUC described by Hand and Till [32]. ROC: receiver operating characteristic, AUC: area under the ROC curve

**Fig. 4:**
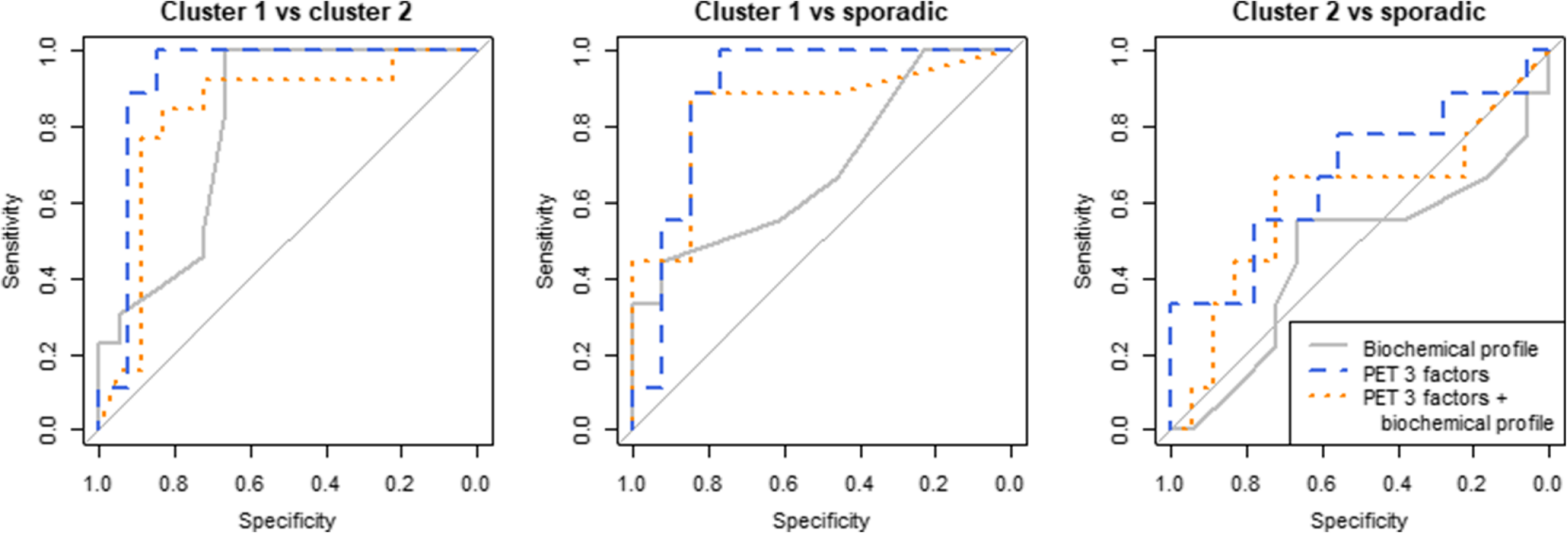
ROC curves for the biochemical profile alone (grey, solid), PET three-factor model (blue, dashed) and the PET model combined with the biochemical profile (orange, dotted) between clusters (cluster 1 vs 2, cluster 1 vs sporadic, cluster 2 vs sporadic) as determined by stratified five-fold multinomial logistic regression and the multiclass AUC described by Hand and Till [32]. ROC: receiver operating characteristic, AUC: area under the ROC curve

The biochemical profile alone could identify the genetic clusters with a mean multiclass AUC of 0.60 (Table 2). The SUV_max_ alone reached a multiclass AUC of 0.85, with a perfect AUC for distinguishing cluster 1 from cluster 2 PPGLs of 1.00 (Fig. 3). The model with three PET factors showed a slightly improved classification performance with a multiclass AUC of 0.88. The three-factor PET model also showed the highest test AUCs for distinguishing sporadic PPGLs from both cluster 1 and 2 PPGLs (Fig. 3). The (multiclass) AUCs for the model with three PET/CT factors were lower (multiclass AUC: 0.81). The addition of the biochemical profile to the imaging model (SUV_max_, three PET factors or three PET/CT factors) increased the (multiclass) AUCs in the training sets but decreased the (multiclass) AUCs in the test sets (Fig. 4). Cluster 1 and 2 PPGLs can be separated with AUCs close to 1.00, cluster 1 and sporadic PPGLs can be distinguished with AUCs around 0.9 and cluster 2 and sporadic PPGLs can be distinguished with AUCs around 0.7. In the sham experiment, no model yielded a (multiclass) AUC different from 0.5 (range: 0.48–0.52).

**Table 2:** Mean (multiclass) AUCs, averaged over 5 folds, between clusters of PPGLs for models based on the biochemical profile, the SUV_max_, the three PET factors, the three PET/CT factors, the three PET features and the imaging variables combined with the biochemical profile, for the training and the test set. The highest mean (multiclass) AUCs in the test set are marked in bold

	Multiclass AUC		AUC cluster 1 vs cluster 2		AUC cluster 1 vs sporadic		AUC cluster 2 vs sporadic	
	Training	Test	Training	Test	Training	Test	Training	Test
Biochemical profile	0.77	0.60	0.90	0.83	0.75	0.55	0.66	0.41
SUV_max_	0.85	0.85	0.98	**1.00**	0.90	0.88	0.67	0.68
+ biochemical profile	0.88	0.81	0.99	0.99	0.90	0.84	0.75	0.60
PET 3 factors	0.91	**0.88**	0.99	0.98	0.93	0.93	0.79	**0.72**
+ biochemical profile	0.97	0.84	0.99	0.95	0.99	0.90	0.92	0.67
PET/CT 3 factors	0.88	0.81	1.00	0.98	0.92	0.85	0.71	0.59
+ biochemical profile	0.93	0.79	1.00	0.95	0.94	0.83	0.85	0.59
PET 3 features	0.89	0.86	0.99	**1.00**	0.96	**0.95**	0.71	0.63
+ biochemical profile	0.94	0.81	1.00	0.98	0.98	0.94	0.84	0.51

*AUC* area under the receiver operating characteristic curves

For the PET and PET/CT model, dimensionality reduction retained three factors in every training set (i.e. 32 lesions in all training sets, Table 3). The PET/CT factors corresponded best to the SUV_max_, ldCT tumour diameter (3D) and ldCT entropy. The retained factors in the PET model corresponded best to SUV_max_, tumour diameter (3D) and grey-level cooccurrence matrix (GLCM) cluster shade. For reproducibility and explainability of the model, these three features were incorporated in a feature-based model, approximating the factor-based model. The feature-based model shows a lower performance than the factor-based model with multiclass AUCs of 0.86 and 0.88 and AUCs for the discrimination of cluster 2 and sporadic PPGLs of 0.63 and 0.72 for the feature-based and factor-based model, respectively (Table 3).

**Table 3:** Results of dimensionality reduction and classification performance of PET and PET/CT radiomic models

	PET	PET/CT
KMO, median (range)^a^	0.96 (0.96–0.96)	0.97 (0.97–0.98)
Cumulative variance all factors, median (range)^a^	0.57 (0.56–0.59)	0.42 (0.40–0.43)
Retained factors^b^	1. Entropy (first order, also closely related to SUV_max_, *ρ* = 0.91) 2. Tumour diameter (3D) 3. Cluster shade (GLCM)	1. SUV_max_ 2. Tumour diameter (3D) on ldCT 3. Entropy on ldCT

*KMO* Kaiser-Meier Olkin measure, *GLCM* grey-level cooccurrence matrix, *ldCT* low-dose CT

^a^The KMO and the cumulative variance of all factors are expressed as median and range of all 5-folds

^b^One factor was retained per ten subjects in the training set

**Fig. 5:**
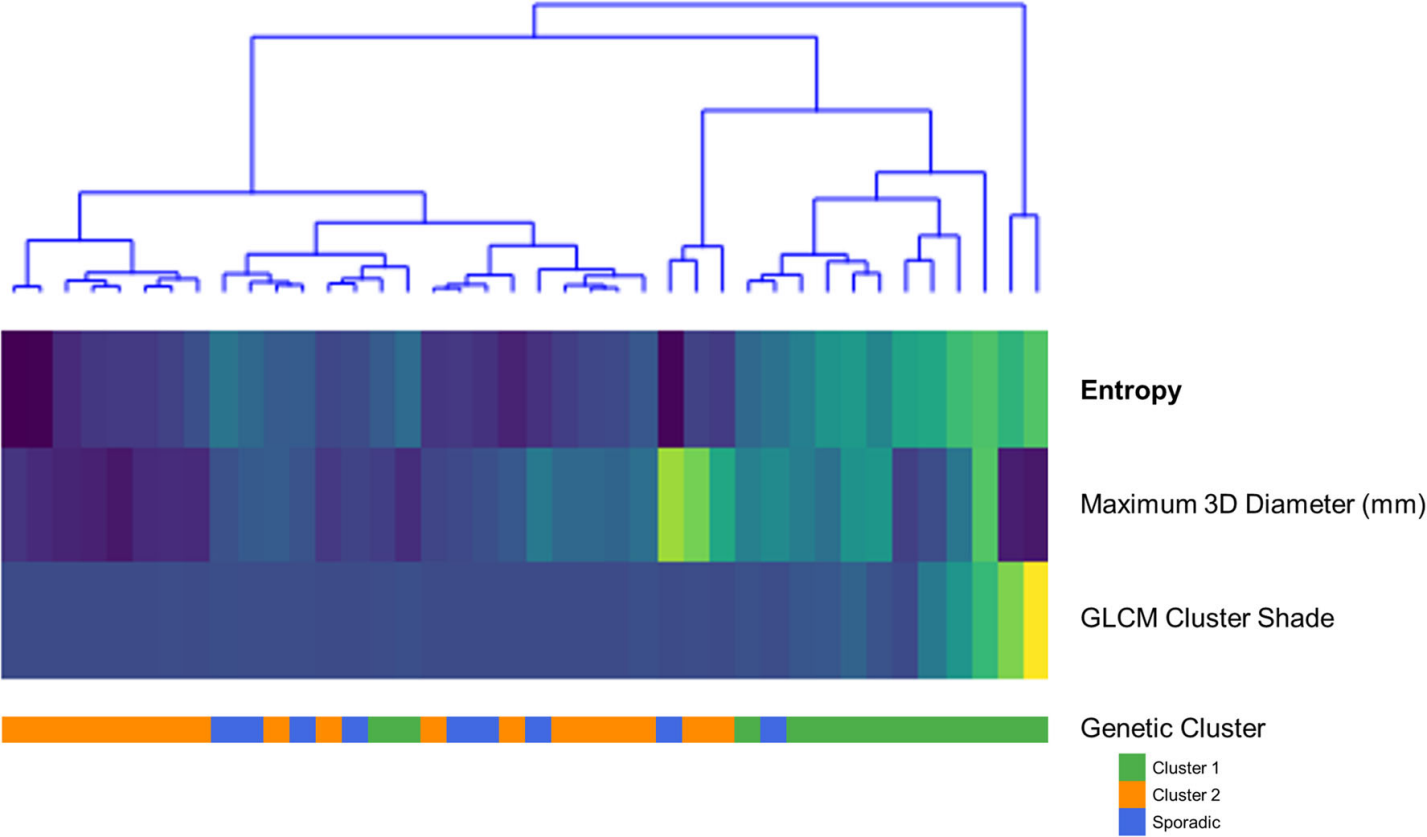
Heatmap of the three PET radiomic features (first-order entropy, maximum 3D diameter, GLCM cluster shade) on the y-axis and the PPGLs on the x-axis, clustered by the PPGLs. Cluster 1, cluster 2 and sporadic PPGLs are represented in blue, red and green, respectively. Features were scaled (centred around 0, variance of 1) to avoid that features with the largest scale would dominate the analysis

**Fig. 6:**
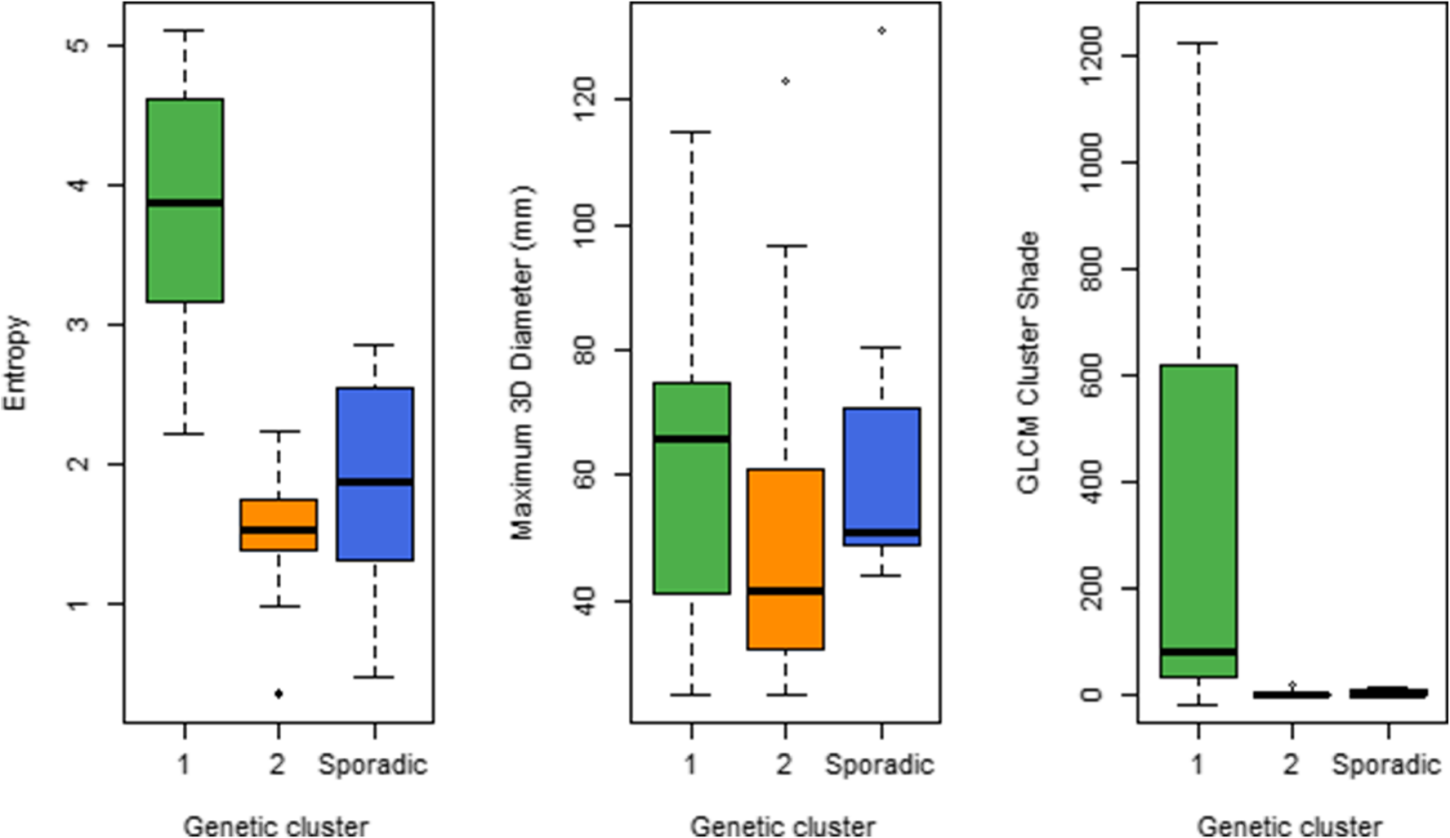
Box plots of selected radiomic features (entropy, maximum 3D diameter and GLCM cluster shade) for cluster 1 (green), cluster 2 (orange) and sporadic (blue) PPGLs

Based on both the PET factors and PET features, cluster 1 PPGLs can be distinguished best from the other clusters (Fig. 5, Fig. 6). Cluster 1 PPGLs show higher means for all features compared to cluster 2 and sporadic PPGLs. Sporadic PPGLs showed imaging characteristics similar to cluster 2 and, to a lesser extent, cluster 1 PPGLs, complicating the differentiation.

## Discussion

In this study, we assessed the added value of radiomic features derived from PET and non-contrast-enhanced ldCT for the characterisation of the genetic cluster of PPGLs. In our previous research, we showed that the SUV_max_ alone could already distinguish hereditary cluster 1 and 2 PPGLs with an AUC of 0.91 (95% CI: 0.80–1.00) [11]. This study focused on the identification of both hereditary and somatic cluster 1 and cluster 2 PPGLs, and sporadic PPGLs in a cross-validated radiomic approach. Our findings demonstrate that SUV_max_ alone already distinguishes cluster 1 and 2 PPGLs with high certainty, but the distinction of clusters 1 and 2 from sporadic PPGLs can be improved moderately by PET radiomics.

Interpretation of the radiomic factors could provide insight into the semantics or tumour phenotype as captured by a PET scan. The first PET factor corresponded to first-order entropy, specifying the randomness in imaging values. Cluster 1 PPGLs show higher mean entropy than cluster 2 and sporadic PPGLs. Entropy is strongly correlated with SUV_max_. Genotype-related changes in energy metabolism have been studied using dynamic [^18^F]FDG-PET/CT scanning [11]. The glucose metabolic rate, phosphorylation rate, vascular blood fraction and SUV_max_ were all significantly higher in cluster 1 than in cluster 2 and/or sporadic PPGLs. This might be associated with increased expression of hexokinase, which indicates an increase in aerobic glycolysis. The second factor corresponded to the 3D tumour diameter. Cluster 1 PPGLs are typically larger than cluster 2 and, to a lesser extent, sporadic PPGLs. The third factor corresponded to GLCM cluster shade, a feature that measures the skewness of the cooccurrence matrix, thereby characterising the tendency of voxel clusters with similar grey levels. A higher cluster shade implies less clustering and therefore more heterogeneous uptake patterns. Cluster 1 PPGLs show higher cluster shade values than sporadic and cluster 2 PPGLs.

In accordance with the findings of Eisenhofer et al [34], our study showed that the biochemical profile alone could distinguish cluster 1 and 2 PPGLs. However, the biochemical profile could not identify sporadic PPGLs with high certainty. Also, the addition of biochemistry to the imaging models did not improve the classification performance, both showing difficulties differentiating sporadic PPGLs. The training set AUCs were increased, compared to a decrease in the test sets. This indicates overfitting of the models, which can be attributed to the total number of six variables in the combined model, disregarding the criterion of 1 variable per 10 subjects in this small dataset [14]. Also, the addition of ldCT features in dimensionality reduction did not improve the performance of the model. This might indicate that the image quality of the ldCT images was insufficient or the images did not contain characteristics suitable for the differentiation of PPGLs. Differently, the addition of the 105 ldCT features almost doubled the total number of features in the dataset, thereby enlarging the feature space and adding new information, contributing to different factors.

The PET factor model was the best-performing model, but differences with the SUV_max_ model were small. Therefore, a simplified model with only the SUV_max_ might be preferred in terms of clinical usability, weighing model performances and the laborious radiomic analysis. Nevertheless, the distinction of sporadic PPGLs from both cluster 1 and 2 PPGLs might be moderately improved by a radiomic model. Cluster 1 and 2 PPGLs generally present distinctive characteristics that can be captured using [^18^F]FDG-PET imaging. Some of these characteristics can even be assessed visually, like [^18^F]FDG uptake and tumour size. Sporadic PPGLs, however, appear more heterogeneous, frequently resembling cluster 2 PPGLs and occasionally resembling cluster 1 PPGLs.

Radiomic research in PPGLs on [^18^F]FDG-PET/CT is limited. Ansquer et al [17] published an article on radiomics in 52 pheochromocytomas with results more or less similar to ours. They also report a higher SUV_max_ in cluster 1 pheochromocytomas than in cluster 2 and pheochromocytomas without germline mutation. In addition, a model with the features metabolic tumour volume and two texture features could identify germline mutation status with an AUC of 0.95. It is challenging to directly compare these results to ours. Ansquer et al [17] included only pheochromocytomas, which were not tested for somatic mutations, i.e. their sporadic group might have included patients with somatic cluster 1 and 2 mutations, while somatic mutations might have impacted the [^18^F]FDG uptake as well. In addition, supervised feature selection was performed on the complete dataset, selecting the features with the best association with the outcome measure, and in the final step, 4-fold cross-validation was performed using these selected features. Besides radiomics, proton (^1^H) nuclear magnetic resonance spectroscopy has been investigated for the identification of genetic clusters of paraganglioma, and the detection of succinate was found to be a highly specific and sensitive hallmark of SDHx mutations [35].

Our study has several strengths and limitations. A strength is that PET/CT images were acquired and reconstructed in accordance with EANM guidelines [20]. The use of these EARL-compliant reconstructions leads to a larger number of reliable, repeatable and reproducible radiomic features [36]. Likewise, radiomic feature extraction was performed and reported conforming to the IBSI recommendations and guidelines, and the TRIPOD statement [21, 22]. Also, unsupervised feature selection, or dimensionality reduction, was performed. In contrast to a supervised approach, dimensionality reduction is not based on the discriminative value of a feature for outcome, but takes into account the interaction of features among themselves and multicollinearity, through which it prevents overfitting of the model [37]. Additionally, dimensionality reduction was incorporated on the training sets per fold instead of on the dataset as a whole, preserving independent test sets. Furthermore, we chose a factor-based over a feature-based approach for the generalisability of our model. Factor analysis was performed incorporated in the folds and instead of selecting features corresponding to the factors, the factors were used as model input. Patterns in corresponding features were compared between folds. In a feature-based approach, insight into these patterns would be limited due to the selection of different features in every fold. In this way, the factor-based approach might advance the generalizability and interpretability of the model and it might provide insight in the semantics or underlying tumour biology of the factors [38]. For the mathematical explainability and reproducibility in the setting of external validation, the PET factor-based model was approximated by a feature-based model. Lastly, we performed a sham experiment to validate our findings [33].

The main limitation of the study is the small sample size. Sample size calculation for radiomics is practically infeasible, as the required number of patients depends on, among other things, the strength of the biological signal, the homogeneity of the data and the complexity of the mathematical model [28]. Orlhac et al [28] state that the minimal sample size is around 70 lesions, in the case of a biological signal that is well reflected by the radiomic features investigated in a cross-validation approach. Our population was smaller than the recommended 70 patients but was unique in terms of patient population. To make the best use of this unique PPGL cohort, we have performed stratified 5-fold cross-validation with strict dimensionality reduction that prevented multicollinearity and retained only one factor for every ten patients in the training set, thereby reducing the overfitting of the model [14].

In conclusion, PET radiomics achieves a better identification of PPGLs compared to biochemistry, SUV_max_ and/or ldCT radiomics, especially for the differentiation of sporadic PPGLs. However, a model with only SUV_max_ might be preferred, weighing model performances against the laborious radiomic analysis. Radiomics could only mildly improve the overall clinical classification performance for PPGL, warranting external validation in a larger cohort to validate our findings.

## Supplementary information

ESM 1 (DOCX 78 kb)

ESM 2 (XLSX 124 kb)

## Abbreviations

[^18^F]FDG: 2-[^18^F]fluoro-2-deoxy-D-glucose
AUC: Area under the receiver operating characteristic curve
CT: Computed tomography
EANM: European Association of Nuclear Medicine
GLCM: Grey-level cooccurrence matrix
IBSI: Image Biomarker Standardisation Initiative
KMO: Kaiser-Meier-Olkin
ldCT: Low-dose computed tomography
MAX: Myc-associated factor X
NF1: Neurofibromatosis type 1
PPGL: Pheochromocytoma and paraganglioma
RET: Rearranged during transfection
SDHA/B/C/D/AF2: Succinate dehydrogenase subunits A, B, C, D and assembly factor 2
SUV: Standardised uptake value
TMEM127: Transmembrane protein 127
VHL: Von Hippel-Lindau
VOI: volume of interest

## Declarations

### Guarantor

The scientific guarantor of this publication is Floris H. P. van Velden, Leiden University Medical Center, Leiden, the Netherlands.

### Statistics and Biometry

One of the authors has significant statistical expertise.

### Study subjects or cohorts overlap

Some study subjects or cohorts have been previously reported in [10, 11, 18].

### Methodology

• Retrospective

• Diagnostic or prognostic study

• Performed at one institution
