# Supplementary material for: [18F]FDG-PET/CT radiomics for the identification of genetic clusters in pheochromocytomas and paragangliomas
Source: Eur Radiol. 2022 Aug 24;32(10):7227–36. doi: 10.1007/s00330-022-09034-5 (PMC9474528; doi:10.1007/s00330-022-09034-5)
Supplement: Supplementary file 1 — (DOCX 78 kb) [file 330_2022_9034_MOESM1_ESM.docx]

**Supplementary File 1: [^18^F]FDG PET/CT radiomics for the identification of genetic clusters in pheochromocytomas and paragangliomas**

**Image Biomarker Standardisation Initiative Reporting Guidelines [1]**

| **Patient** | | | | | |
| --- | --- | --- | --- | --- | --- |
| Volumes of interest | Pheochromocytomas and paragangliomas (PPGLs) | | | | |
| Patient preparation   - Patient instructions - Drugs - Equipment | Patients fasted for at least 6 hours before imaging and were orally hydrated with 500 mL of water. | | | | |
| Radioactive tracer   - Tracer - Administration method - Injected activity - Uptake time prior to acquisition - Competing substances | PET acquisition was started 60 (55-75) minutes after intravenous administration of [^18^F]FDG in an antecubital vein, using a non-linear dosage (D) regimen:   - <95 kg body weight (BW): D = 1.7*BW (4 min per bed position) - >95 kg BW: D = 0.017778*W² (4 min per bed position) - 3 min/bed position: D*1.333 - 2 min/bed position: D*2 - Maximum: 555 MBq, minimum: 20 MBq   Serum glucose levels were below 8.0 mmol/L. | | | | |
| Contrast agent | NA | | | | |
| Comorbidities | Patients with diabetes mellitus were included as long as fasted serum glucose was below 8.0 mmol/L and no short-acting insulins were given 4 hours prior to injection of FDG. | | | | |
| **Acquisition** | | | | | |
| Acquisition protocol | A low-dose CT (ldCT) was performed, followed by a static PET scan, from the base of the skull to the mid-thigh, approximately 60 minutes post-injection. | | | | |
| Scanner type | Siemens Biograph 40 mCT (Siemens Healthineers) | | | | |
| Imaging modality | PET/CT | | | | |
| Static/dynamic scans | Static | | | | |
| Scanner calibration | All PET/CT scanners were regularly cross-calibrated with the dose calibrator of the hospital pharmacy and the dose calibrator of the pharmacy that delivers the [^18^F]FDG according to EARL-accreditation and guidelines for quality control of the Dutch Association of Nuclear Medicine (NVNG). | | | | |
| Patient instructions | Free-breathing PET scans were acquired with the patient instructed not to move and positioned in restraining/supportive devices. | | | | |
| Anatomical motion correction | No anatomical motion correction is performed, due to the hybrid nature of PET/CT it is assumed that the position of PET and ldCT with respect to the scanner’s coordinate system stays constant. | | | | |
| Scan duration | 3 or 4 min/bed position, dose adjusted accordingly | | | | |
| Tube voltage ldCT | 100-140 kVp | | | | |
| Tube current ldCT (median (range)) | 104 (70-145) mA | | | | |
| Time-of-flight | Yes | | | | |
| **Reconstruction** | | | | | |
| In plane resolution | PET: 3.18×3.18 mm²  ldCT: 0.64×0.64 – 1.27×1.27 mm² | | | | |
| Image slice thickness | PET: 3 mm  ldCT: 3 mm | | | | |
| Image slice spacing | PET: 3 mm  ldCT: 3mm | | | | |
| Convolution kernel and exposure (median (range)) | B31f  52 (35-72) mAs | | | | |
| Reconstruction method | High-definition ordered-subsets expectation maximation with 3 iterations and 21 subsets with time of flight | | | | |
| Point spread function modelling | Point spread function-based | | | | |
| Image corrections   - Attenuation correction - Other corrections | Attenuation correction based on ldCT. Correction for scatter, randoms, normalization, dead time and physical decay was applied. | | | | |
| **Image processing – data conversion** | | | | | |
| SUV normalisation | Body weight | | | | |
| Other data conversions | NA | | | | |
| **Image processing – post acquisition processing** | | | | | |
| Anti-aliasing | NA | | | | |
| Noise suppression | NA | | | | |
| Post-reconstruction smoothing filter | 8 mm full width at half maximum (FWHM) 3D Gaussian | | | | |
| Intensity normalisation | NA | | | | |
| Other post-acquisition processing methods | NA | | | | |
| **Segmentation** | | | | | |
| Segmentation method   - Method - Number of experts, expertise, consensus strategies - Settings - Images | VOIs were delineated semi-automatically using 3DSlicer (www.slicer.org) and in-house built software implemented in Python 3.7 (Python Software Foundation).  VOI were delineated on the [^18^F]FDG-PET scans using an isocontour that applies a threshold of 41% of the peak standardized uptake value (SUV_peak_), corrected for local background [3].  The PET-IndiC extension of 3DSlicer was used to delineate a spacious box around the tumour, excluding surrounding tissues with high [^18^F]FDG-avidity. In case of high serum levels of catecholamines it is known that brown adipose tissue (BAT) uptake can be strongly increased [4]. As BAT can be present in the perirenal fossa, it could interfere with delineation of the pheochromocytoma. Patients with pheochromocytomas and high BAT uptake in the perirenal fossa were excluded (N=2). The SUV_peak_, obtained using a sphere of 12 mm in diameter [2], was determined in PET-IndiC extension. In-house built software implemented in Python was used to calculate the background uptake and define the volume of interest, as described by Frings et al. [3]. Lesions were excluded when the minimal size recommendation of 64 voxels per VOI was not met (N=1) [27]. Regions of central necrosis, which were not included by the adaptive threshold algorithm, were not manually added to the VOI, since in PPGL, the predictive performance of the radiomic model is not affected by the addition of areas of central necrosis [5]. LdCT images were delineated manually slice-by-slice (axial).  VOIs were delineated by WN (PhD candidate on PET radiomics with 4 years of experience) and supervised by DV (nuclear medicine physician with 13 years of experience). | | | | |
| Conversion to mask | NA | | | | |
| **Image processing – image interpolation** | | | | | |
| Interpolation algorithm   - Algorithm - Interpolation grid - Dimensions - Extrapolation | PET images were not interpolated,  ldCT images were interpolated to isotropic voxels using B-spline interpolation, with grids aligned by the input origin and only covering the VOI (PyRadiomics default). | | | | |
| Interpolated voxel dimensions | PET: NA  ldCT: 1.5×1.5×1.5 mm³ | | | | |
| **Image processing – ROI interpolation and re-segmentation** | | | | | |
| Interpolation algorithm | NA | | | | |
| Partially masked voxels | NA | | | | |
| Re-segmentation methods | NA | | | | |
| **Image processing – discretisation** | | | | | |
| Discretisation method   - Method - Number of bins/bin size - Lowest intensity first bin | Discretisation using a fixed bin size. Bin edges were equally spaced from 0 (e.g. 0-0.5, 0.5-1, etc) and the lowest grey value was discretized into the first bin.  PET: 0.5 g/mL  ldCT: 25 HU | | | | |
| **Image processing – image transformation** | | | | | |
| Image filter | NA | | | | |
| **Image biomarker computation** | | | | | |
| Biomarker set (PyRadiomics nomenclature, if IBSI nomenclature differed, it was added in brackets) | PET and ldCT:   - First Order Statistics (18 features): 10^th^ Percentile, 90^th^ Percentile, Energy, Entropy (Intensity Histogram Entropy), Interquartile Range, Kurtosis, Maximum, Mean Absolute Deviation, Mean, Median, Minimum, Range, Robust Mean Absolute Deviation, Root Mean Squared, Skewness, Total Energy (not present in IBSI definitions), Uniformity (Intensity histogram uniformity), Variance - Shape based (14 features): Elongation, Flatness, Least Axis Length, Major Axis Length, Maximum 2D Diameter Column, Maximum 2D Diameter Row, Maximum 2D Diameter Slice, Maximum 3D Diameter, Mesh Volume (Volume), Minor Axis Length, Sphericity, Surface Area, Surface Volume Ratio, Voxel Volume (Approximate Volume) - Grey Level Cooccurrence Matrix (GLCM; 24 features): Autocorrelation, Joint Average, Cluster Prominence, Cluster Shade, Cluster Tendency, Contrast, Correlation, Difference Average, Difference Entropy, Difference Variance, Joint Energy (Angular Second Moment), Joint Entropy, Informational Measure of Correlation 1, Informational Measure of Correlation 2, Inverse Difference Moment, Inverse Difference Moment Normalized, Inverse Difference, Inverse Difference Normalized, Inverse Variance, Maximum Probability (Joint Maximum), Sum Entropy, Sum of Squares (Joint Variance), Sum Average, Maximal Correlation Coefficient - Grey Level Run Length Matrix (GLRLM; 16 features): Short Run Emphasis, Long Run Emphasis, Grey Level Non-Uniformity, Grey Level Non-Uniformity Normalized, Run Length Non-Uniformity, Run Length Non-Uniformity Normalized, Run Percentage, Grey Level Variance, Run Variance, Run Entropy, Low Grey Level Run Emphasis, High Grey Level Run Emphasis, Short Run Low Grey Level Emphasis, Short Run High Grey Level Emphasis, Long Run Low Grey Level Emphasis, Long Run High Grey Level Emphasis - Grey Level Size Zone Matrix (GLSZM; 16 features): Small Area Emphasis (Small Zone Emphasis), Large Area Emphasis (Large Zone Emphasis), Grey Level Non-Uniformity, Grey Level Non-Uniformity Normalized, Size-Zone Non-Uniformity (Zone Size Non-Uniformity), Size-Zone Non-Uniformity Normalized (Zone Size Non-Uniformity Normalized), Zone Percentage, Grey Level Variance, Zone Variance (Zone Size Variance), Zone Entropy (Zone Size Entropy), Low Grey Level Zone Emphasis, High Grey Level Zone Emphasis, Small Area Low Grey Level Emphasis (Small Zone Low Grey Level Emphasis), Small Area High Grey Level Emphasis (Small Zone High Grey Level Emphasis), Large Area Low Grey Area Emphasis (Large Zone Low Grey Level Emphasis), Large Area High Grey Level Emphasis (Large Zone High Grey Level Emphasis) - Grey Level Dependence Matrix (GLDM; 14 features): Small Dependence Emphasis (Low Dependence Emphasis), Large Dependence Emphasis (High Dependence Emphasis), Grey Level Non-Uniformity, Dependence Non-Uniformity (Dependence Count Non-Uniformity), Dependence Non-Uniformity Normalized (Dependence Count Non-Uniformity Normalized), Grey Level Variance, Dependence Variance (Dependence Count Variance), Dependence Entropy (Dependence Count Entropy), Low Grey Level Emphasis (Low Grey Level Count Emphasis), High Grey Level Emphasis (High Grey Level Count Emphasis), Small Dependence Low Grey Level Emphasis (Low Small Dependence Low Grey Level Emphasis), Small Dependence High Grey Level Emphasis (Low Dependence High Grey Level Emphasis), Large Dependence Low Grey Level Emphasis (High Dependence Low Grey Level Emphasis), Large Dependence High Grey Level Emphasis (High Dependence Low Grey Level Emphasis) - Neighbouring Grey Tone Difference Matrix (NGTDM; 5 features): Coarseness, Contrast, Busyness, Complexity, Strength   PET:   - Total Lesion Glycolysis | | | | |
| IBSI compliance | Yes | | | | |
| Robustness | Regions of central necrosis, which were not included by the adaptive threshold algorithm, were not manually added to the VOI, since in PPGL, the predictive performance of the radiomic model is not affected by the addition of areas of central necrosis [5]. | | | | |
| Software availability | PyRadiomics 3.0 in Python 3.7 (Python Software Foundation) | | | | |
| **Image biomarker computation – texture parameters** | | | | | |
| Texture matrix aggregation | GLCM and GLRLM: 3D: average; GLSZM, GLDM and NGTDM: 3D | | | | |
| Distance weighting | No weighting | | | | |
| Cooccurrence matrix symmetry | Symmetric | | | | |
| Cooccurrence matrix distance | Chebyshev distance of 1 | | | | |
| Size zone matrix linkage distance | Chebyshev distance of 1 | | | | |
| Distance zone matrix linkage distance | NA | | | | |
| Distance zone matrix distance norm | NA | | | | |
| Neighbouring grey tone difference matrix distance | Chebyshev distance of 1 | | | | |
| Grey level dependence matrix distance | Chebyshev distance of 1 | | | | |
| Grey level dependence matrix coarseness | 0 | | | | |
| **Machine learning and radiomic analysis** | | | | | |
| Diagnostic and prognostic modelling | Documented in the next section using the Transparent Reporting of a multivariable prediction model for Individual Prognosis or Diagnosis (TRIPOD) Checklist: Prediction Model Development and Validation [6]. | | | | |
| Comparison with known factors | Radiomic models were compared with the biochemical profile (adrenergic, noradrenergic, dopaminergic) and the SUV_max_. | | | | |
| Multicollinearity | Unsupervised dimension reduction using redundancy filtering (Spearman correlation matrix threshold = 0.95) and factor analysis was performed in the folds. Features were scaled (centered around 0, variance of 1) to avoid that features with the largest scale would dominate the analysis. Redundancy filtering was performed by creating a correlation matrix of all features. For each row, the number of times a feature exhibits an absolute correlation above the threshold (≥0.95) is collected in a vector. The feature with the most absolute correlations exceeding the threshold is represented by most other features and is thus removed. A new correlation matrix is generated with the remaining features and the process is iteratively repeated until the generated correlation matrix does not contain absolute correlations above the threshold. Factor analysis, a method to project the feature space on a lower-dimensional latent meta-feature (i.e., factor) space, was performed on the redundancy filtered correlation matrix using an orthogonal rotation. In this way, the first factor explained the largest possible variance in the dataset and succeeding factors explained the largest variance in orthogonal directions. The sampling adequacy of the model was determined by the Kaiser-Meier-Olkin (KMO) measure, which was predefined to be ≥0.9. One factor was selected for every ten subjects in the training set. Dimensionality reduction was performed in FMradio (Factor Modeling for Radiomics Data) in R [7]. | | | | |
| Model availability | The models generated during and/or analysed during the current study are available from the corresponding author on reasonable request. | | | | |
| Data availability | The datasets generated during and/or analysed during the current study are available from the corresponding author on reasonable request. | | | | |
| **TRIPOD checklist** | | | | | |
| Version | October 1, 2020 | | | | |
| Objectives | Evaluate whether [^18^F]FDG-PET/CT radiomics alone and combined with the biochemical profile could predict the genetic cluster of PPGLs. | | | | |
| Source of data | Patients with PPGL with a known mutation status and who underwent a [^18^F]FDG PET/CT scan in the Radboud University Medical Center between 2011 and 2018 were retrospectively included. A selection of these patients has previously been studied [5; 8; 9]. This retrospective database study has been reviewed and approved by the Commission on Medical Research Involving Human Subjects Region Arnhem-Nijmegen, the Netherlands (protocol code: 2018-4655, date of approval: 10 December 2018). Informed consent was waived because of the retrospective nature of the study. Patients that objected to the use of their anonymised data were excluded. | | | | |
| Participants | Forty PPGLs with known germline or somatic mutation status and who underwent a [^18^F]FDG PET/CT scan following the EANM-guidelines were included in the radiomic analysis [10]. Patient characteristics are described in table 1 of the manuscript. | | | | |
| Outcome | Genetic cluster based on germline and somatic mutations. | | | | |
| Predictors | Radiomic features as extracted from the [^18^F]FDG PET and ldCT scans (specified above). Unsupervised dimension reduction using redundancy filtering and factor analysis was performed. For the PET factor-based model, the factors corresponded best to entropy, tumour diameter (3D) and cluster shade (GLCM). For the PET/CT factor-based model, the factors corresponded best to SUV_max_, tumour diameter (3D) on ldCT and entropy on ldCT. Predictors in the PET feature-based model were entropy, tumour diameter (3D) and cluster shade (GLCM). Also, a model with only SUV_max_ was assessed. Performance of the imaging models was assessed with and without the biochemical profile | | | | |
| Statistical analysis | Stratified five-fold multinomial logistic regression for the prediction of the genetic clusters of PPGLs was performed. The dataset was split into five equal-sized folds, stratified for the genetic clusters. Each subgroup consecutively served as a test set and the remaining four-fifths of patients was used as the training set. In each fold, dimensionality reduction of the radiomic feature set of the training set was performed using redundancy filtering and factor analysis. The factors were used as independent variables in multinomial logistic regression. Predictive performances were presented as mean multiclass AUCs and mean AUCs between clusters as determined over the five folds for the training and test sets [11]. Models were trained for the SUV_max_, PET factors, PET/CT factors and for imaging features combined with the biochemical profile (adrenergic, noradrenergic, dopaminergic).  A sham experiment was conducted to validate the findings [12]. The outcome labels (genetic cluster) were randomly shuffled for 100 iterations and mean AUCs were calculated for both the training and the test set. Randomisation of the outcome labels preserves the distributions and multicollinearity of the radiomic features and the prevalence of the outcome, but it uncouples their hypothesised relation. | | | | |
| Model performance, test (multiclass) AUCs |  | Multiclass AUC | AUC cluster 1 vs cluster 2 | AUC cluster 1 vs sporadic | AUC cluster 2 vs sporadic |
|  | Biochemical profile | 0.60 | 0.83 | 0.55 | 0.41 |
|  | SUV_max_ | 0.85 | 1.00 | 0.88 | 0.68 |
|  | SUV_max_ + biochemical profile | 0.81 | 0.99 | 0.84 | 0.60 |
|  | PET (3 factors) | 0.88 | 0.98 | 0.93 | 0.72 |
|  | PET (3 factors) + biochemical profile | 0.84 | 0.95 | 0.90 | 0.67 |
|  | PET/CT (3 factors) | 0.81 | 0.98 | 0.85 | 0.59 |
|  | PET/CT (3 factors) + biochemical profile | 0.79 | 0.95 | 0.83 | 0.59 |
|  | PET (3 features) | 0.86 | 1.00 | 0.95 | 0.63 |
|  | PET (3 features) + biochemical profile | 0.81 | 0.98 | 0.94 | 0.51 |

**References**

1 Zwanenburg A, Leger S, Vallieres M, Lock S (2019) Image biomarker standardisation initiative - feature definitions v11. DOI: 10.48550/arXiv.1612.070032 Wahl RL, Jacene H, Kasamon Y, Lodge MA (2009) From RECIST to PERCIST: Evolving Considerations for PET Response Criteria in Solid Tumors. J Nucl Med 50:122S-150S

3 Frings V, van Velden FHP, Velasquez LM, Hayes W, al. e (2014) Repeatability of Metabolically Active Tumor Volume Measurements with FDG PET/CT in Advanced Gastrointestinal Malignancies: A Multicenter Study. Radiology 273:539-548

4 Steinberg JD, Vogel W, Vegt E (2017) Factors influencing brown fat activation in FDG PET/CT: a retrospective analysis of 15,000+ cases. Br J Radiol. DOI: 10.1259/bjr.20170093.5 Noortman WA, Vriens D, Mooij CDY et al (2021) The Influence of the Exclusion of Central Necrosis on [18F]FDG PET Radiomic Analysis. Diagnostics (Basel) 11:1296

6 Moons KG, Altman DG, Reitsma JB et al (2015) Transparent Reporting of a multivariable prediction model for Individual Prognosis or Diagnosis (TRIPOD): explanation and elaboration. Ann Intern Med 162:W1-73

7 Peeters CF, Übelhör C, Mes SW et al (2019) Stable prediction with radiomics data. DOI: 10.48550/arXiv.1903.11696

8 van Berkel A, Vriens D, Visser EP et al (2019) Metabolic Subtyping of Pheochromocytoma and Paraganglioma by (18)F-FDG Pharmacokinetics Using Dynamic PET/CT Scanning. J Nucl Med 60:745-751

9 van Berkel A, Rao JU, Kusters B et al (2014) Correlation between in vivo 18F-FDG PET and immunohistochemical markers of glucose uptake and metabolism in pheochromocytoma and paraganglioma. J Nucl Med 55:1253-1259

10 Boellaard R, O'Doherty MJ, Weber WA et al (2010) FDG PET and PET/CT: EANM procedure guidelines for tumour PET imaging: version 1.0. Eur J Nucl Med Mol Imaging 37:181-200

11 Hand DJ, Till RJ (2001) A Simple Generalisation of the Area Under the ROC Curve for Multiple Class Classification Problems. Machine Learning 45:171-186

12 Buvat I, Orlhac F (2019) The dark side of radiomics: on the paramount importance of publishing negative results. J Nucl Med 60:1543-1544
